# Dynamics of the human bile acid metabolome during weight loss

**DOI:** 10.1038/s41598-024-75831-1

**Published:** 2024-10-28

**Authors:** Andreas Schmid, Gerhard Liebisch, Ralph Burkhardt, Hannah Belikan, Sebastian Köhler, Daniel Steger, Leonie Schweitzer, Jörn Pons-Kühnemann, Thomas Karrasch, Andreas Schäffler

**Affiliations:** 1 Basic Research Laboratory of Molecular Endocrinology, Adipocyte Biology and Biochemistry, University of Giessen, Giessen, Germany; 2 Institute of Clinical Chemistry and Laboratory Medicine, University of Regensburg, Regensburg, Germany; 3 Department of Internal Medicine – Endocrinology, Diabetology, Metabolism, University of Giessen, Giessen, Germany; 4 Medical Statistics, Institute of Medical Informatics, University of Giessen, Giessen, Germany; 5 Department of Internal Medicine, Giessen University Hospital, Klinikstrasse 33, 35392 Giessen, Germany

**Keywords:** Obesity, Bile acids, Bariatric surgery, Low calory diet, Weight loss, Metabolome, Roux-en-Y gastric bypass, Liver, Metabolomics

## Abstract

Bile acids (BA) are supposed to cause metabolic alterations after bariatric surgery (BS). Here we report the longitudinal dynamics of the human BA metabolome by LC–MS/MS after BS versus low calory diet (LCD) in two obesity cohorts over 12 months. Rapid and persistent oscillations of 23 BA subspecies could be identified with highly specific patterns in BS vs. LCD. TCDCA, GLCA, and TLCA represent most promising candidates for drug development.

## Introduction

Bile acids (BA) play an outstanding role in dietary lipid emulsification, gut microbiome signature, brown adipose tissue thermogenesis, and metabolism of lipids, hepatic cholesterol or carbohydrates^1–5^. Systemic BA were suggested to act as peripheral hormones^6^ on white adipose tissue and the term “*bilokines*” was subsequently introduced^7^. This term conceptualizes the hypothesis that BA circulating in peripheral blood (in contrast to portal vein) target specific nuclear receptors (e.g. FXRα) or transmembrane receptors (e.g. TGR5) in peripheral tissues such as adipose tissue^6–9^. Bariatric surgery (BS) such as Roux-en-Y gastric bypass (RYGB)^10,11^ represents the most effective long-term treatment of obese patients and exerts early and beneficial metabolic effects^1,6,12,13^. Besides incretin hormones derived from entero-endocrine cells of the small intestine such as GLP-1 and GIP^14–16^, BA^17–19^ are promising candidates^1,12^ that could mediate the effects of RYGB^7^.

There are numerous studies in humans and mice that documented increased concentrations of BA upon RYGB^1,2,12,20–29^. Although subtypes of BA were demonstrated to increase upon BS^1,30–32^, only total BA or conjugated *versus* non-conjugated BA were investigated in most studies^1,2^. Thus, alterations ^18,30–36^ of systemic BA after RYGB are poorly characterized and studies on human BA concentrations in obesity or during weight loss are contradictory and difficult to interpret. Cohorts were small, different in study design, and did not compare BS with low caloriy diet (LCD) longitudinally. Different methods of quantification have been used and studies did not distinguish between BA concentrations on one hand, and percentage composition of BA within a changing and flexible pool of BA (total BA pool; that is the concentration of all BA subspecies). TUDCA, GUDCA, UDCA, GHCA, GCDCA, GUDCA were reported to increase upon RYGB, whereas taurine-conjugated BA were occasionally shown to decrease; CA, CDCA, DCA, GDCA, and HCA were reported to remain elevated 2 years after BS^1,2,30,37^.

According to the *bilokine* hypothesis^7^, each single BA should be measured by high end techniques such as LC–MS/MS upon BS in order to obtain reliable data and to identify single druggable BA with physiological impact. The human BA metabolome has not yet been monitored systematically and longitudinally in obesity and during weight loss by LC–MS/MS.The present study deciphers the oscillations of 16 single BA species (and 7 classes of BA) by LC–MS/MS in obesity and longitudinally during weight loss induced by RYGB vs. LCD in two large and deeply characterized cohorts of patients. Specific patterns of BA alterations are presented as a basis and data resource for addressing single BA as potential drug targets in obesity and associated metabolic diseases.

## Results

### Short- and long-term effects of RYGB versus LCD on body weight

The present study cohorts of patients undergoing RYGB versus LCD represent two carefully selected subgroups of patients retrieved from the perpetual *ROBS* study^38^ (please find detailed information within extended data file 5). For the present study on BA metabolome, only data sets of patients who completed all visits (V0, V3, V12) over 1 year of follow-up were selected. Only bariatric patients undergoing RYGB (but not vertical sleeve gastrectomy) and patients on LCD were investigated. Table 1 shows the characteristics of the study population undergoing extensive BA metabolome analysis. These both cohorts comprise 91 patients undergoing RYGB and 88 patients undergoing LCD. The multi-professional and standardized LCD program consisted of three phases over 52 weeks (months 1–3: only liquid and very low calory diet allowed by ingestion of a specified formula diet 5 times/day with a maximum of 800 kcal/day; months 4–5: transition phase with gradual replacement of liquid diet by mixed, low-fat, low calory meals together with ongoing life style modification; months 6–12: stabilization phase with low calory diet by normal meals and sustained life style modification). The study was approved by the local ethical committee at the University of Giessen, Germany (registration number: *AZ 101/14*). All patients gave informed and written consent and were informed about the aim of the study.

**Table 1:** Characteristics of the study populations.

RYGB (n = 91)			
Parameter	V0	V3	V12
Sex females n (%) males n (%)	75 (82.4%) 16 (17.6%)		
Age (years ± SD); [range]	40.8 ± 10.5 [18 – 60]		
Body weight (kg)	149.1 ± 24.0	121.1 ± 19.5 *	94.8 ± 16.2 *
BMI (kg/m^2^)	51.8 ± 5.3	42.1 ± 4.5 *	33.1 ± 4.1*
Weight loss (kg)		− 27.9 ± 6.0	− 54.2 ± 16.2
Weight loss (kg; %)		− 18.7 ± 2.4	− 36.0 ± 7.8
Weight loss (BMI)		− 9.7 ± 1.6	− 18.7 ± 5.3
Weight loss (BMI; %)		− 18.7 ± 2.4	− 35.8 ± 8.0
LCD (n = 88)			
Parameter	V0	V3	V12
Sex females n (%) males n (%)	59 (67.0%) 29 (33.0%)		
Age (years ± SD); [range]	42.4 ± 11.6 [20 – 67]		
Body weight (kg)	128.8 ± 23.1	105.2 ± 18.8 *	99.1 ± 19.3 *
BMI (kg/m^2^)	43.4 ± 5.6	35.5 ± 5.5 *	33.5 ± 5.8 *
Weight loss (kg)		− 23.5 ± 9.7	− 29.7 ± 15.3
Weight loss (kg; %)		− 18.1 ± 6.4	− 22.6 ± 9.8
Weight loss (BMI)		− 7.9 ± 2.9	− 9.9 ± 4.7
Weight loss (BMI; %)		− 18.2 ± 6.6	− 22.7 ± 9.7

*RYGB* roux-en-Y gastric bypass, *LCD* low calory diet, *V* visit (months), *BMI* body mass index.

Mean ± SD is shown. * *P* < 0.001 vs. V0).

### Dynamics of glycine-conjugated and taurine-conjugated bile acids after RYGB

**Fig. 1:**
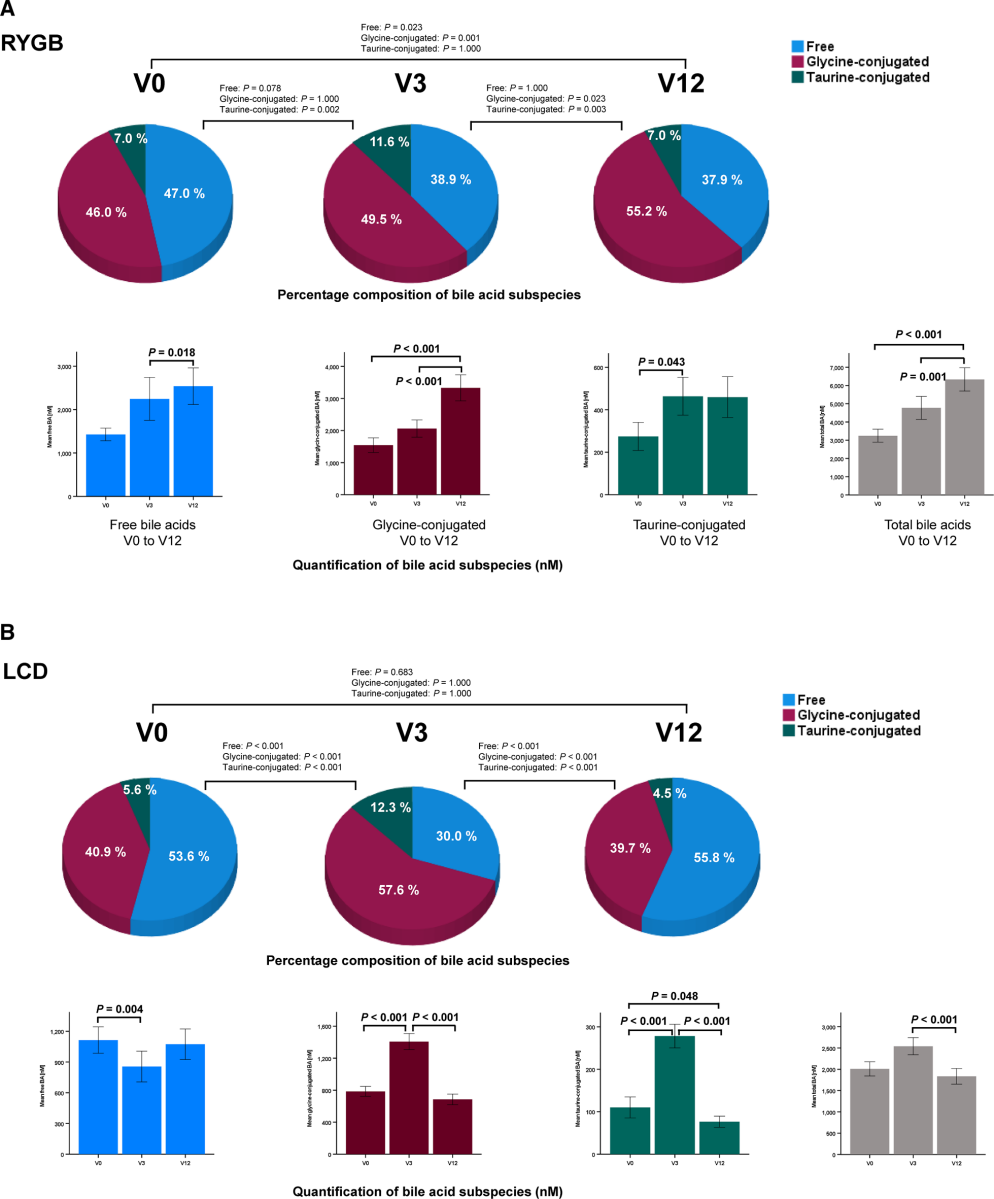

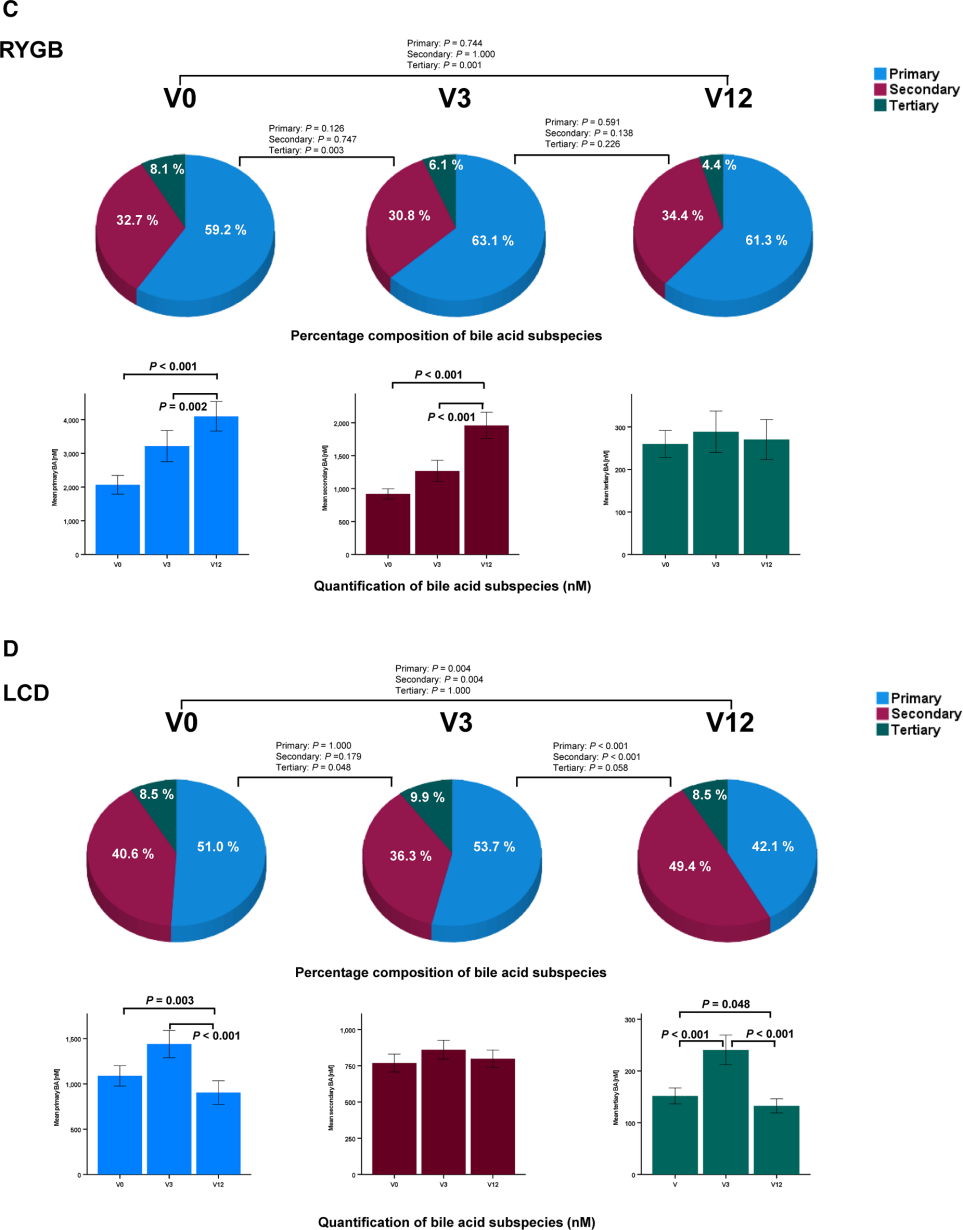

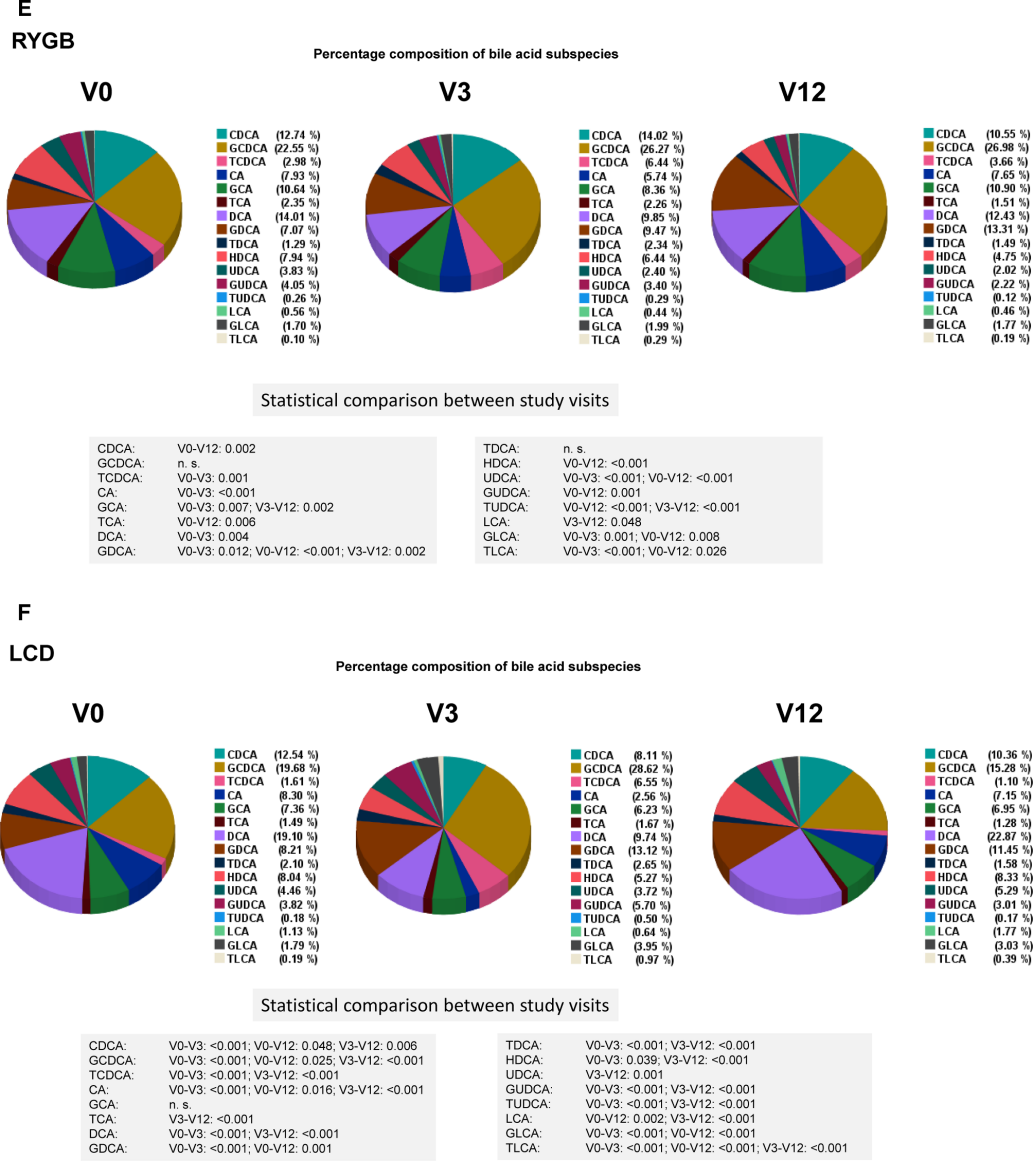
Oscillations and dynamics of the human BA metabolome in obesity before and during weight lowering therapies by RYGB and LCD. Percentage composition (upper panels, pie charts) and concentration (lower panels, bar diagrams) of BA subspecies in n = 91 patients (75 females, 16 males) before (V0) and after RYGB (V3: 3 months; V12: 12 months) is presented. Percentage is relatively to the total BA pool that increases continuously after RYGB. Percentage composition (upper panels, pie charts) and concentration (lower panels, bar diagrams) of BA subspecies in n = 88 patients (59 females, 29 males) before (V0) and during LCD (V3: 3 months; V12: 12 months) is presented. From V0 to V3, patients were only allowed to ingest a very low calory and liquid formula diet. From V3 to V12, patients switched to a normal hypo-caloric diet. Percentage is relatively to the total BA pool that increases transiently at V3. The absolute concentrations of BA (nM) were measured by LC–MS/MS. The Friedman`s two way analysis of variance by ranks was used for related samples and *P* values were corrected for multiple testing according to Bonferroni´s method. RYGB, Roux-en-Y gastric bypass; LCD, low calory diet; V, visit (months); BA, bile acids (**A**,** B**) Free and conjugated BA before and after RYGB versus LCD in obese patients. (**C**,** D**) Primary, secondary and tertiary BA before and after RYGB versus LCD in obese patients. (**E**,** F**) Percentage composition of 16 single BA before and after RYGB versus LCD in obese patients. The concentration of each single BA (nM) was measured by LC–MS/MS and is given in the extended data files 1 and 2.

After RYGB, percentage (Fig. 1A, upper panel) and absolute concentration (Fig. 1A, lower panel) of glycine-conjugated BA significantly increased in a stepwise manner from V0 to V12. Whereas the percentage of taurine-conjugated BA decreased, their absolute concentrations significantly increased. This observation is conceivable, since the total BA pool strongly increased from 3249 ± 3359 at V0 up to 6332 ± 6084 nM at V12 together with an increase of free BA concentrations (Fig. 1A, lower panel). A strong, stepwise and consistent increase of total and free BA with rising concentrations of glycine-conjugated and – to a less extent – of taurine-conjugated BA characterizes the dynamics of BA after RYGB.

### Dynamics of glycine-conjugated and taurine-conjugated bile acids during LCD

During LCD, percentage (Fig. 1B, upper panel) and absolute concentration (Fig. 1B, lower panel) of glycine-conjugated BA significantly increased from V0 to V3 and returned to pre-study levels at V12. The percentage of taurine-conjugated BA transiently increased at V3 and their absolute concentrations transiently increased at V3 as well. In contrast to RYGB, alterations of BA during LCD are of transient nature. Total and conjugated BA are increased at V3, whereas free BA are decreased.

### Dynamics of primary, secondary and tertiary bile acids after RYGB.

After RYGB, percentage of primary and secondary BA subspecies did not change significantly, whereas the percentage of tertiary BA (UDCA, GUDCA, TUDCA) decreased (Fig. 1C, upper panel). In contrast to these relative changes, the absolute concentrations of primary and secondary BA subspecies strongly increased (Fig. 1C, lower panel) whereas tertiary BA remained unchanged in the context of rising total and free BA. A stepwise and strong increase of primary and secondary but not tertiary BA characterizes the dynamics of BA after RYGB.

### Dynamics of primary, secondary and tertiary bile acids during LCD.

During LCD, percentage (Fig. 1D, upper panel) and concentrations of primary BA (Fig. 1D, lower panel) were significantly lower at V12 when compared to pre-study levels. Secondary BA had a higher percentage at V12 whereas their absolute concentrations remained unchanged during LCD over 12 months. Relative percentage of tertiary BA showed only marginal variation. However, the absolute concentration of tertiary BA significantly increased at V3 and returned to pre-study levels at V12. Primary BA are lower after 12 months of LCD, whereas tertiary BA are transiently increased at V3.

### Quantification and longitudinal monitoring of 16 single human bile acids before and after RYGB

**Fig. 2:**
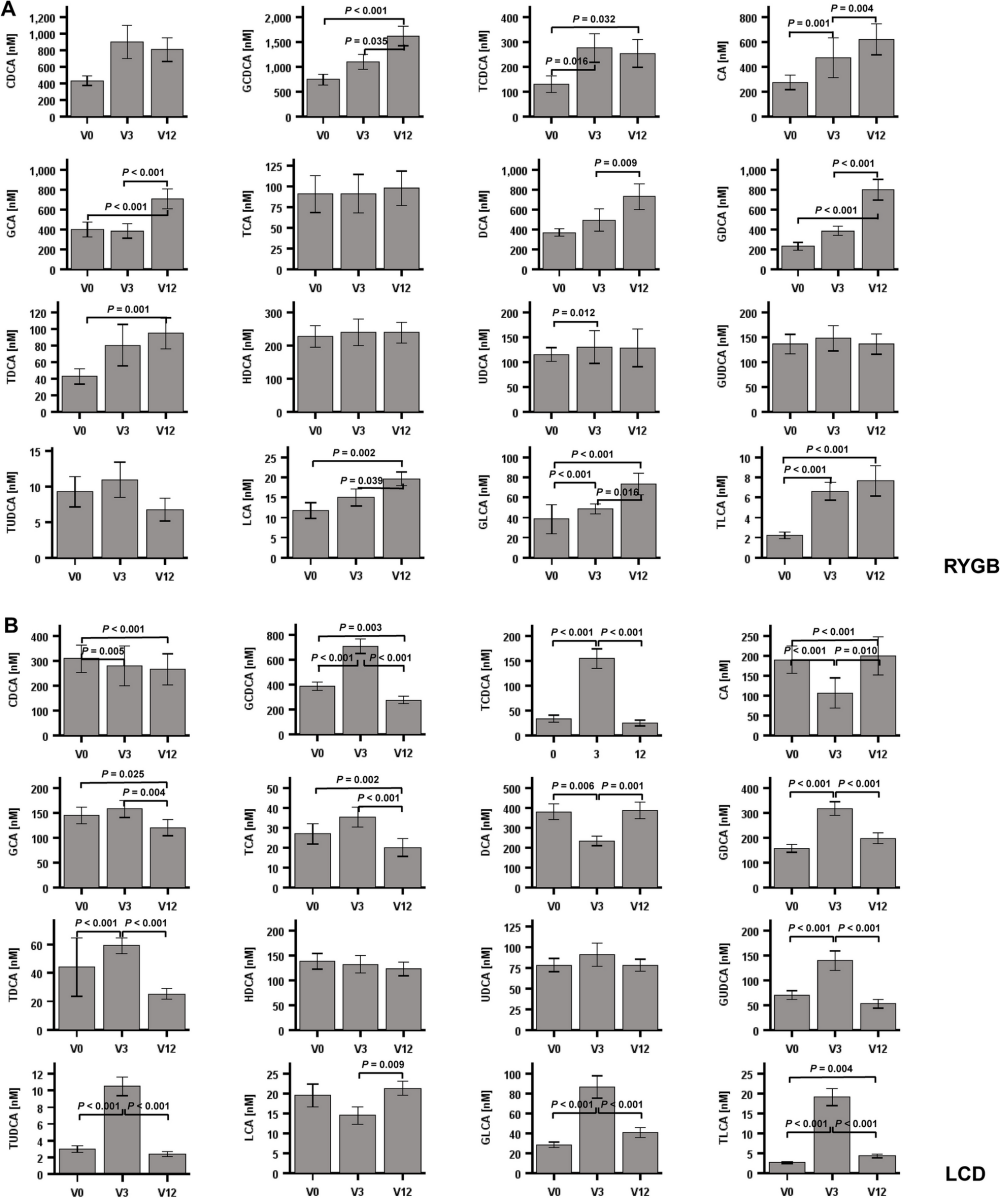
Nanomolar concentrations of 16 human bile acids (BA) by LC–MS/MS after Roux-en-Y gastric bypass (RYGB) (panel A) and during low calory diet (LCD). Mean ± SEM is presented by the bar diagrams. The Friedman`s two way analysis of variance by ranks was used for related samples and *P* values were corrected for multiple testing according to Bonferroni´s method.

Percentage composition and longitudinal alteration of 16 single BA species after RYGB and LCD over one year is summarized in Fig. 1 E, F. Statistical evaluation of the respective nanomolar concentrations is summarized in Fig. 2 A and B. For practical reasons and as a resource, the absolute concentrations in each single patient are presented to the readership by extended data files 1 and 2. Taken together, due to their significant increases, the bile acids CA, DCA, UDCA, LCA, TCDCA, TDCA, TLCA, GCA, GCDCA, GDCA and GLCA represent promising candidates mediating the metabolic effects of bariatric surgery.

### Quantification and longitudinal monitoring of 16 single human bile acids during LCD

The percentage of 16 human bile acid subspecies was monitored longitudinally before and after LCD (Fig. 1F). Only GCA showed no variation during LCD, the exact changes and significances are shown in Fig. 1F. However, since the total bile acid pool underlies variations during the time course of LCD, the absolute concentrations are of higher interest and are summarized in Fig. 2B. Only UDCA and HDCA remained unchanged during LCD. As expected, most variations could be observed at V3, since patients were on liquid hypocaloric formula diet until V3 and were then switched to hypocaloric diet using normal meals. Most bile acids (TCDCA, TDCA TUDCA, TLCA, GCDCA, GDCA, GUDCA, GLCA) showed an upregulation at V3 followed by a decrease to pre-study levels. Some bile acids (CA, DCA) showed the opposite regulation, a decrease at V3 followed by an increase. Some bile acids were longitudinally downregulated (CDCA, TCA, GCA). Taken together, ingestion of solely a liquid hypocaloric formula diet over 3 months characteristically upregulates a broad variety of bile acids (mainly derivates of CA and DCA) transiently. Other species (unconjugated CA and DCA) show the opposite phenomenon. After replacement of the liquid diet by solid diet, these dynamics were reversible. Remarkably, relative weight loss was nearly identical in RYGB and LCD after 3 months (V3) (expressed as percentage loss of weight and BMI, Table 1). These data indicate a similar extent of catabolism in the RYGB and LCD groups from V0 to V3. However, weight loss significantly continued in RYGB for 12 months, cumulating in a loss of 36% of original body weight, whereas in LCD, weight stabilized after a gradual switch back to regular diet from liquid formula diet. Thus, continued catabolism might be responsible for persistent alterations of BA after RYGB. Surprisingly, diet-induced catabolism over three months is able to cause similar effects on bile acids. In conclusion, catabolism per se induces BA alterations independent of the surgical procedure.

## Discussion

In the literature, increases of total and primary bile acids after RYGB have been reported repetitively in humans^7,39^. Among taurine-conjugated bile acids, TUDCA, as well as several glycine-conjugated bile acids, has been shown to increase after RYGB in humans^7^. However, a systematic and longitudinal quantification by LC–MS/MS of all human bile acid subspecies with absolute concentrations and relative (percentage) changes has not yet been available so far. Of major interest for potential drug development, CA, UDCA, TCDCA, TLCA, and GLCA were identified to increase rapidly after RYGB. Of these, only TCDCA, GLCA and TLCA remained increased over 12 months. Even more remarkable, among all single BA species, only TCDCA, GLCA and TLCA fullfill the following three criteria simultaneously: a) rapid increase after RYGB, b) persistent increase after RYGB, c) rapid increase after LCD during the very low calory phase with ingestion of liquid diet only. Thus, these three BA should be selected for evaluation of their potential pharmacological effects in obesity, adipocyte biology, and metabolism. The present biostatistical data on single bile acids measured by LC–MS/MS might provide a basis for the future development of human reference ranges. However, as typical for clinical cohorts we observed a high grade of inter-individual variability of bile acids. This represents a limitation of the study and might lead to the definition of relatively wide normal ranges.

**Fig. 3:**
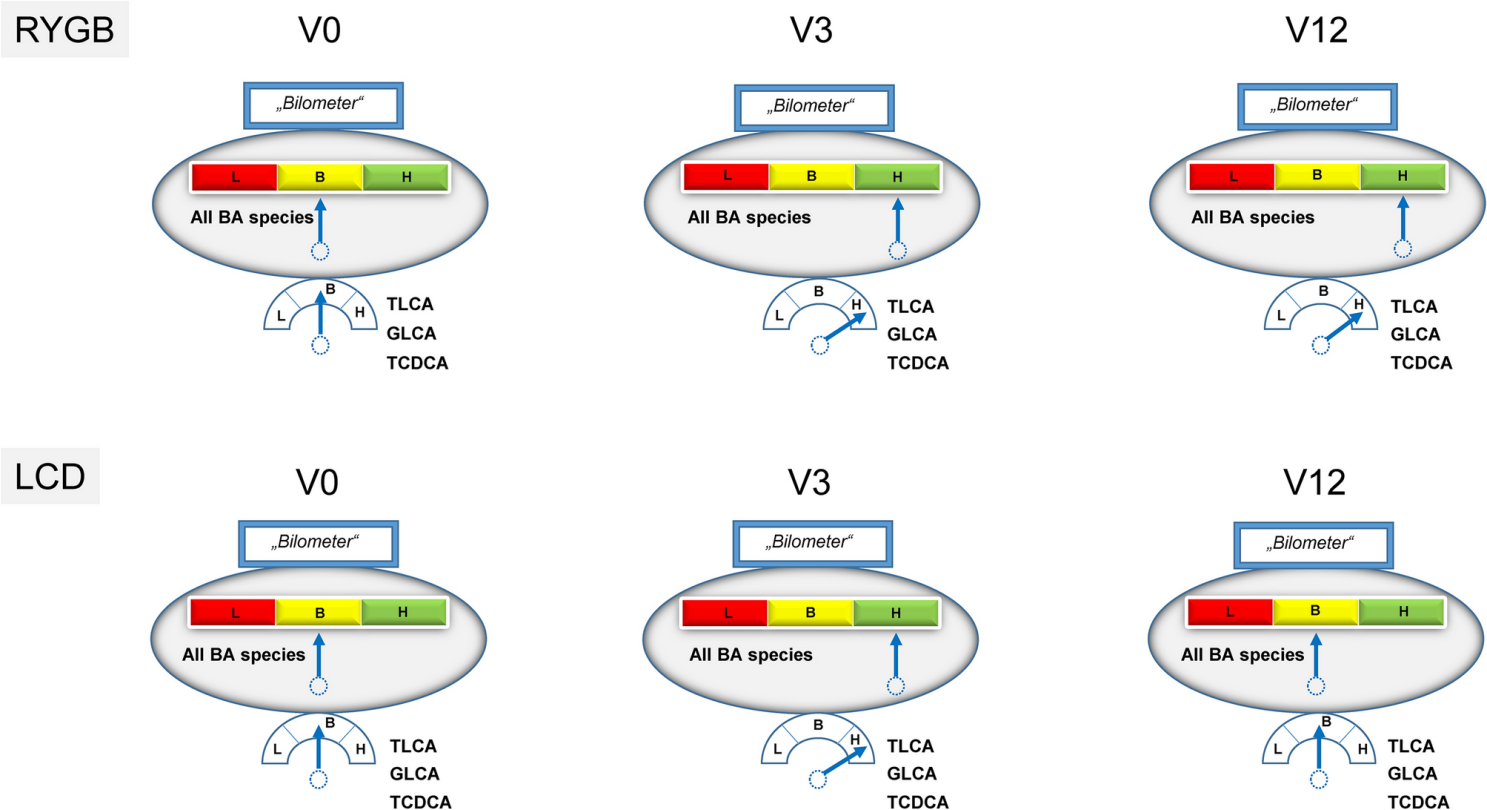
Characteristic patterns of BA metabolome oscillations during weight-lowering therapies in obese patients. The large “*Bilometer*” scales summarize overall and longitudinal oscillations of BA main species (total, free, taurine-/glycine-conjugated, primary, secondary) during RYGB versus LCD in n = 179 obese patients. RYGB causes rapid and persistent increases of BA concentrations. Surprisingly, a very low calory and strictly liquid diet over three months mimics the effects of RYGB at V3 but has no persistent effects on BA metabolome. The small “*Bilometer*” scales indicate the identified and specific alterations of TLCA, GLCA, TCDCA. RYGB, Roux-en-Y gastric bypass; LCD, low calory diet; V, visit (months); BA, bile acids; L, low; B, basal state at V0; H, high. For identification of 16 single BA at each time point in both cohorts, please refer to extended data files 1 and 2.

CA and UDCA represent BA that are exclusively upregulated by RYGB and might therefore account for specific effects of BS. In conclusion, we could identify two characteristic patterns of BA metabolome oscillations (Fig. 3). Upon RYGB, the levels of BA species (total, free, taurine-/glycine-conjugated, primary, secondary) increase early, dramatically, and remain elevated up to one year. In contrast, LCD shows a distinct pattern with rising BA species during the first phase of LCD when patients are set completely on a very low calory and strictly liquid formula diet (V3). In a retrospective chinese study of 37 obese and type 2 diabetic patients undergoing RYGB^39^, the authors found significantly higher concentrations of CDCA at baseline in those individuals who experienced diabetes remission after surgery. Moreover, percentage CDCA was higher in obese and diabetic patients when compared to non-diabetic patients in a cross-sectional analysis. In the cited study^39^, CDCA concentrations significantly decreased one year after surgery in the remission group whereas they remained unchanged in the non-remission group. This report is in accordance with our observation (Fig. 1E) that percentage CDCA significantly (p = 0.002) decreases from V0 (12.74%) to V12 (10.55%) after RYGB. However, in 91 patients after RYGB we could not demonstrate a significant decrease of absolute CDCA concentrations (Fig. 2A). In contrast, relative (Fig. 1F) and absolute CDCA concentrations significantly decreased in our patients after LCD (Fig. 2B). Most surprisingly, this very low calory and liquid diet over three months mimics the effects of RYGB at V3 but has no long-lasting effects on BA metabolome. When LCD continues but liquid meals are being replaced by normal meals, BA return to pre-study levels. TCDCA, GLCA and TLCA are promising drug targets in obesity-related disorders whereas CA and UDCA seem to be related specifically to RYGB. However, there are some limitations of the study. Grade of weight loss, differing dietary patterns, macro- and micro-nutrient balance, gut microbiome, and environmental factors might have influenced systemic bile acid compositions. We could present characteristic patterns of bile acid species after RYGB vs. LCD by a longitudinal observation. However, it remains unclear whether weight loss and/or caloric restriction primarily caused bile acid alterations or whether the rise of (single) bile acids caused the weight loss. Future molecular and mechanistic studies e.g. in adipocytes are necessary to prove a role of TCDCA, GLCA and TLCA in mediating metabolic changes after RYGB and/or LCD. Toxicity might be a major issue when suggesting bile acid derivates as potential drug targets. It has to be considered that the degree of toxic effects of BA depends on the BA species, the dose/concentration and on the target cell type. For example, BA circulate in the portal vein in very high concentrations, much higher than in the peripheral circulation. Thus, liver cells and bile duct cells are able to tolerate much higher concentrations than for example adipocytes. In a recent study^8^, we could demonstrate that adipocytes can tolerate only micro-molar doses of bile acids such as cholic acid (CA; 100 µM), glycocholic acid (GCA; 100 µM), taurocholic acid (TCA; 100 µM), deoxycholic acid (DCA; 10 µM), glycodeoxycholic acid (GDCA; 10 µM), taurodeoxycholic acid (TDCA; 10 µM), or chenodeoxycholic acid (CDCA; 10 µM). Higher doses in the milli-molar range are toxic to adipocytes. The physiological concentrations in the systemic circulation are ranging in the non-toxic nano-molar range, as clearly shown by the present study. As is known, derivates of lithocholic acid are higly toxic whereas ursodeoxycholic acid is non-toxic and has already been used as a therapeutic target in clinical medicine. In the present study, the toxic LCA is present in the peripheral circulation after RYGB in a range of only 11.7 ± 18.6 to 19.6 ± 16.3 nM (< < 1% of bile acid pool. However, when taking toxicity of BA into account, bile acid derivates must be administered only subcutaneously or intravenously within the physiological nano-molar range in order to avoid high and toxic doses via the oral/intestinal route.

## Methods

### Bile acid metabolome quantification

Bile acids (BA) were quantified using liquid chromatography tandem mass spectrometry (LC–MS/MS), following the method described by Krautbauer et al. ^40^ and based on the principles outlined in Krautbauer et al.^41^ and Scherer et al.^42^. Briefly, 100 μL of patient material, quality controls (QCs), calibrators, and internal standard blank (water) were placed in a 1.5 mL Eppendorf tube. Then, 20 μL of internal standard mix containing stable isotope labelled BA for all analytes except for HDCA and its conjugates was added. To increase extraction efficiency, 30 µL of 1 mol/L HCl was added and proteins were precipitated with 1 mL of acetonitrile. The dried supernatants were then redissolved in 100 μL of methanol/water (30/70, v/v). The LC–MS/MS system consisted of a 1200 series binary pump, a 1200 series isocratic pump (Agilent, Waldbronn, Germany), an HTC Pal autosampler (CTC Analytics, Zwingen, Switzerland), and a hybrid triple quadrupole linear ion trap mass spectrometer API 4000 Q-Trap (Applied Biosystems, Ontario, Canada) operated in negative ESI mode. The LC analysis was performed using a NUCLEOSHELL RP18 core–shell column (Machery & Nagel, Düren, Germany) with dimensions of 50 mm × 2 mm and a particle size of 2.7 μm. The mobile phase consisted of methanol/water (1/9, v/v) as mobile phase A and methanol as mobile phase B, both containing 0.1% ammonium hydroxide (25%) and 10 mmol/L ammonium acetate (pH 9). The gradient used was 80% A for 0.05 min, followed by a stepwise linear decrease to 53% A at 0.1 min, 49% A at 2.0 min, and 28% A at 4.5 min. The flow rate was set to 0.5 mL/min. A column wash was performed using 100% B for 0.5 min, followed by re-equilibration using 100% A for 0.6 min at a flow rate of 0.8 mL/min. BA were monitored by selected reaction monitoring using a product ion of *m/z* 74 for glyco-conjugated, *m/z* 80 for tauro-conjugated and the precursor ion for free BA species. Quantification is based on the ratio of area counts of the analyte to its respective stable isotope internal standard. HDCA species were related to their respective UDCA. Calibration was performed using six levels generated by BA addition to charcoal-stripped and pooled serum from healthy donors. The abbreviations of bile acid subspecies are given here: Primary bile acids: CA, cholic acid; CDCA, chenodeoxycholic acid. Secondary bile acids: DCA, deoxycholic acid, HDCA, hyodeoxycholic acid. LCA, lithocholic acid. Tertiary bile acids: UDCA, ursodeoxycholic acid. Taurine-conjugated bile acids: TCA, taurocholic acid; TCDCA, taurochenodeoxycholic acid; TDCA, taurodeoxycholic acid; TLCA, taurolithocholic acid; TUDCA, tauroursodeoxycholic acid. Glycine-conjugated bile acids: GCA, glycocholic acid; GCDCA, glycochenodeoxycholic acid; GDCA, glycodeoxycholic acid; GLCA, glycolithocholic acid; GUDCA, glycoursodeoxycholic acid, GHDCA, glycohyodeoycholic acid. Derivates of hyodeoxycholic acid (THDCA, taurohyodeoxycholic acid and GHDCA, glycohyodeoycholic acid) were under the detection limit of LC–MS/MS.

### Data base handling and biostatistics

As described earlier^38^, the *ROBS* database was programmed with *FileMaker Pro 13*, a relational database management program which runs on Windows and Mac Systems as a multi-user system. An additional web interface can be programmed for database applications on iOs- and android-compatible devices. Pseudonymization runs over a 256 bit encoded database that is separated from the general network. In this system, target appointments for the visits are fixed automatically. A separate database with an individual input mask was programmed for data entry patient visits. For statistical analysis, data were extracted into the computational software program IBM SPSS statistics, version 29.0. The Friedman`s two way analysis of variance by ranks was used for related samples and *P* values were corrected for multiple testing according to Bonferroni´s method (Supplementary information in extended data files 1 and 2).

## Supplementary Information

Supplementary Information 1.

Supplementary Table 1.

Supplementary Table 2.

Supplementary Information 2.

Supplementary Information 3.

Supplementary Information 4.

## Data Availability

Data availability and data on personal request. Each single bile acid concentration at any time point in individual patients can be retrieved from extended data files 3 and 4. On personal and individual request, the authors are able to provide researches with yet unpublished data on correlations of 16 bile acid species with a huge variety of measured parameters in the study cohort. Individual requests can be sent to andreas.schaeffler@innere.med.uni-giessen.de These parameters comprise 15 anthropometric items, 16 clinical chemistry parameters, 8 cytokines/chemokines, and 9 adipokines for each study visit. Together with 16 bile acid species, 7 main classifications of bile acids, and n = 3 visits, n = 50.400 possible correlations of interest can potentially be investigated. In addition, 34 classified socio-economic variables were documented. Moreover, authors can provide associations of bile acid concentrations at V0 with yet unpublished gene expression data in subcutaneous and visceral adipose tissue of patients who underwent RYGB.
