# Supplementary material for: Dynamics of the human bile acid metabolome during weight loss
Source: Sci Rep. 2024 Oct 28;14:25743. doi: 10.1038/s41598-024-75831-1 (PMC11519931; doi:10.1038/s41598-024-75831-1)
Supplement: Supplementary file 1 — Supplementary Information 1. [file 41598_2024_75831_MOESM1_ESM.pdf]

Legends for excel files:

Supplementary data file 3: Resources file (*Excel work sheet*): **Bile acid (BA) metabolome quantification by LC-MS/MS in the *ROBS* study cohort of obese patients undergoing bariatric surgery by RYGB.** The LC-MS/MS quantification data of patients at all study visits are shown together with basal anthropometric parameters. *ROBS*, Research in Obesity and Bariatric Surgery; V, visit; BMI, body mass index;

Supplementary data file 4: Resources file (Excel work sheet): **Bile acid (BA) metabolome quantification by LC-MS/MS in the *ROBS* study cohort of obese patients undergoing low calory diet (LCD).** The LC-MS/MS quantification data of patients at all study visits are shown together with basal anthropometric parameters. *ROBS*, Research in Obesity and Bariatric Surgery; V, visit; BMI, body mass index;
