# Supplementary material for: Dynamics of the human bile acid metabolome during weight loss
Source: Sci Rep. 2024 Oct 28;14:25743. doi: 10.1038/s41598-024-75831-1 (PMC11519931; doi:10.1038/s41598-024-75831-1)
Supplement: Supplementary file 2 — Supplementary Table 1. [file 41598_2024_75831_MOESM2_ESM.pdf]

Quantification of single bile acids by HPLC-MS/MS before and after Roux-en-Y gastric bypass (RYGB)				
**Bile acid species (nmol/l)**	**RYGB V0**	**RYGB V3**	**RYGB V12**	***P*** *Friedman test*
**CA ↑**	274.59 ± 565.06	471.16 ± 1528.29	620.43 ± 1180.54	V0 – V3: 0.001<br>V0 – V12: n. s.<br>V3 – V12: 0.004
**CDCA ↔**	431.60 ± 545.92	898.70 ± 1884.82	806.44 ± 1371.97	V0 – V3: n. s.<br>V0 – V12: n. s.<br>V3 – V12: n. s.
**DCA ↑**	368.41 ± 354.34	493.65 ± 1057.57	727.86 ± 1233.95	V0 – V3: n. s.<br>V0 – V12: n. s.<br>V3 – V12: 0.009
**UDCA ↑**	115.17 ± 131.48	130.03 ± 313.41	128.13 ± 359.85	V0 – V3: 0.012<br>V0 – V12: n. s.<br>V3 – V12: n. s.
**HDCA ↔**	227.25 ± 305.58	239.95 ± 384.35	238.41 ± 292.04	V0 – V3: n. s.<br>V0 – V12: n. s.<br>V3 – V12: n. s.
**LCA ↑**	11.73 ± 18.65	14.96 ± 20.11	19.68 ± 16.33	V0 – V3: n. s.<br>V0 – V12: 0.002

				V3 – V12: 0.039
**TCA ↔**	90.79 ± 213.41	91.00 ± 221.84	97.57 ± 198.90	V0 – V3: n. s.<br>V0 – V12: n. s.<br>V3 – V12: n. s.
**TCDCA ↑**	129.46 ± 319.79	274.87 ± 544.57	253.44 ± 535.36	V0 – V3: 0.016<br>V0 – V12: 0.032<br>V3 – V12: n. s.
**TDCA ↑**	42.81 ± 88.79	80.33 ± 239.63	94.49 ± 180.62	V0 – V3: n. s.<br>V0 – V12: 0.001<br>V3 – V12: n. s.
**TUDCA ↔**	9.27 ± 20.21	10.96 ± 23.71	6.77 ± 15.11	V0 – V3: n. s.<br>V0 – V12: n. s.<br>V3 – V12: n. s.
**TLCA ↑**	2.22 ± 3.11	6.62 ± 8.33	7.67 ± 14.44	V0 – V3: < 0.001<br>V0 – V12: < 0.001<br>V3 – V12: n. s.
**GCA ↑**	400.25 ± 717.73	384.75 ± 701.91	708.10 ± 958.86	V0 – V3: n. s.<br>V0 – V12: < 0.001<br>V3 – V12: < 0.001
**GCDCA ↑**	742.39 ± 1028.03	1096.39 ± 1416.55	1616.26 ± 1868.41	V0 – V3: n. s.<br>V0 – V12: < 0.001

				V3 – V12: 0.035
**GDCA ↑**	229.20 ± 384.63	384.86 ± 451.21	798.27 ± 991.71	V0 – V3: n. s.<br>V0 – V12: < 0.001<br>V3 – V12: < 0.001
**GUDCA ↔**	135.64 ± 188.31	147.72 ± 240.66	135.69 ± 192.97	V0 – V3: n. s.<br>V0 – V12: n. s.<br>V3 – V12: n. s.
**GLCA ↑**	38.35 ± 137.56	48.55 ± 46.97	73.57 ± 101.49	V0 – V3: < 0.001<br>V0 – V12: < 0.001<br>V3 – V12: 0.016

Table 1: Longitudinal quantification of single human bile acid (BA) subspecies measured by HPLC-MS/MS in n=91 obese patients before (V0) and after RYGB (V3, V12). Data are given as mean concentrations ± SD (standard deviation) in nmol/l. For direct comparison of mean BA levels between visits, the non-parametric Friedman test was applied and calculated significance was corrected according to Bonferroni´s correction. ↑ increase, ↓ decrease, ↑↓ rapid increase at V3 followed by decline back to pre-study levels, ↓↑ rapid decrease at V3 followed by an increase at V12, ↔ no change. Please note: Increase/decrease can be different from the percentage shifts of BA (Fig. 1, A-F, upper panels) due to the increase of the total BA pool. V, study visit; V3, after three months; V12, after 12 months; n.s., not significant.
