# Supplementary material for: Dynamics of the human bile acid metabolome during weight loss
Source: Sci Rep. 2024 Oct 28;14:25743. doi: 10.1038/s41598-024-75831-1 (PMC11519931; doi:10.1038/s41598-024-75831-1)
Supplement: Supplementary file 3 — Supplementary Table 2. [file 41598_2024_75831_MOESM3_ESM.pdf]

Quantification of single bile acids by HPLC-MS/MS before and after low calory diet (LCD)				
**Bile acid species (nmol/l)**	**LCD V0**	**LCD V3**	**LCD V12**	***P*** *Friedman test*
**CA** ↓↑	190.26 ± 317.54	106.28 ± 355.38	199.85 ± 452.21	V0 – V3: < 0.001<br>V0 – V12: < 0.001<br>V3 – V12: 0.010
**CDCA** ↓	307.93 ± 512.57	279.75 ± 757.27	264.98 ± 586.48	V0 – V3: 0.005<br>V0 – V12: < 0.001<br>V3 – V12: n. s.
**DCA** ↓↑	380.36 ± 380.37	233.22 ± 226.18	387.38 ± 385.43	V0 – V3: 0.006<br>V0 – V12: n. s.<br>V3 – V12: 0.001
**UDCA** ↔	78.33 ± 75.69	90.81 ± 130.88	77.91 ± 68.22	V0 – V3: n. s.<br>V0 – V12: n. s.<br>V3 – V12: n. s.
**HDCA** ↔	138.20 ± 148.42	131.77 ± 164.32	122.65 ± 130.14	V0 – V3: n. s.<br>V0 – V12: n. s.<br>V3 – V12: n. s.
**LCA** ↑	19.47 ± 27.23	14.47 ± 20.79	21.32 ± 17.00	V0 – V3: n. s.<br>V0 – V12: n. s.

				V3 – V12: 0.009
**TCA ↓**	26.92 ± 48.35	35.29 ± 47.49	20.12 ± 42.22	V0 – V3: n. s.<br>V0 – V12: 0.002<br>V3 – V12: < 0.001
**TCDCA ↑↓**	33.53 ± 64.09	154.27 ± 185.61	24.51 ± 54.91	V0 – V3: < 0.001<br>V0 – V12: n. s.<br>V3 – V12: < 0.001
**TDCA ↑↓**	44.08 ± 193.31	59.19 ± 52.27	25.16 ± 35.45	V0 – V3: < 0.001<br>V0 – V12: n. s.<br>V3 – V12: < 0.001
**TUDCA ↑↓**	3.00 ± 3.67	10.48 ± 10.23	2.41 ± 2.67	V0 – V3: < 0.001<br>V0 – V12: n. s.<br>V3 – V12: < 0.001
**TLCA ↑↓**	2.64 ± 2.79	19.08 ± 20.24	4.30 ± 4.20	V0 – V3: < 0.001<br>V0 – V12: 0.004<br>V3 – V12: < 0.001
**GCA ↓**	144.41 ± 154.27	157.27 ± 159.12	120.22 ± 153.97	V0 – V3: n. s.<br>V0 – V12: 0.025<br>V3 – V12: 0.004
**GCDCA ↑↓**	386.46 ± 320.08	707.71 ± 560.53	274.89 ± 290.73	V0 – V3: < 0.001<br>V0 – V12: 0.003

				V3 – V12: < 0.001
**GDCA ↑↓**	156.33 ± 146.31	317.15 ± 252.38	197.27 ± 199.30	V0 – V3: < 0.001<br>V0 – V12: n. s.<br>V3 – V12: < 0.001
**GUDCA ↑↓**	70.45 ± 80.52	139.43 ± 181.80	52.28 ± 81.16	V0 – V3: < 0.001<br>V0 – V12: n. s.<br>V3 – V12: < 0.001
**GLCA ↑↓**	28.32 ± 26.23	86.55 ± 106.11	40.87 ± 46.66	V0 – V3: < 0.001<br>V0 – V12: n. s.<br>V3 – V12: < 0.001

Table 2: Longitudinal quantification of single human bile acid (BA) subspecies measured by HPLC-MS/MS in n=88 obese patients before (V0) and during LCD (V3, V12). Data are given as mean concentrations ± SD (standard deviation) in nmol/l. For direct comparison of mean BA levels between visits, the non-parametric Friedman test was applied and calculated significance was corrected according to Bonferroni´s correction. ↑ increase, ↓ decrease, ↑↓ rapid increase at V3 followed by decline back to pre-study levels, ↓↑ rapid decrease at V3 followed by an increase at V12, ↔ no change. Please note: Increase/decrease can be different from the percentage shifts of BA (Fig. 1 A-F, upper panels) due to the increase of the total BA pool. V, study visit; V3, after three months; V12, after 12 months; n.s., not significant.
