# Supplementary material for: Dynamics of the human bile acid metabolome during weight loss
Source: Sci Rep. 2024 Oct 28;14:25743. doi: 10.1038/s41598-024-75831-1 (PMC11519931; doi:10.1038/s41598-024-75831-1)
Supplement: Supplementary file 6 — Supplementary Information 4. [file 41598_2024_75831_MOESM6_ESM.pdf]

Extended data file 5: Characteristics of the study population *ROBS* (Research in Obesity and Bariatric Surgery).

Paired serum samples and tissue samples from subcutaneous (abdominal) and visceral (intra-abdominal) adipose tissue were collected by the *ROBS* (*Research in Obesity and Bariatric Surgery*) study cohort. *ROBS* is an open-label, non-randomized, longitudinal, observational (explorative and confirmatory) and perpetual study of obese patients undergoing either bariatric surgery (vertical sleeve gastrectomy [VSG] or Roux-en-Y gastric bypass [RYGB]) or a low calorie formula diet (LCD) in a single tertiary care center. Patients were treated by a multidisciplinary team of physicians and professionals from Internal Medicine, Endocrinology, Diabetology, Visceral Surgery, Psychosomatic Medicine, Psychotherapy, Nutritional Science, and Sports Medicine. All patients gave informed consent and were informed about the aim of the study. Data anonymization and privacy policy were accurately applied. Table 1 summarizes inclusion criteria, exclusion criteria, investigations, monitoring, and data acquisition of the *ROBS* study.

**Inclusion criteria**	Bariatric Surgery (RYGB and VSG):<br>Obese patients with a body mass index (BMI) $\geq$ 40 kg/m^2 or with a BMI $\geq$ 35 kg/m^2 and coexisting type 2 diabetes and/or obesity-related comorbidities.<br>Low calory Diet:<br>Obese patients with a body mass index (BMI) $\geq$ 35 kg/m^2
**Exclusion criteria**	pregnancy, evidence of or suspicion on underlying endocrine diseases, untreated bulimia nervosa and binge eating behavior, use of illicit drugs, neoplasm, severe psychiatric disorders, psychosis, and psychopathologic instability.
**Special investigations in bariatric patients**	Pre-Study (V0):<br>history and physical examination, 2mg dexamethasone suppression test, routine clinical chemistry and endocrinological work-up, 2h-oral glucose tolerance test (OGTT in non-diabetic subjects), gastro-duodenoscopy, abdominal ultrasound, long-term (24h-)blood pressure measurement, 12-lead electrocardiography, obstructive sleep apnea screening, chest X-ray, body plethysmography, bio-impedance analysis, echocardiography, nutritional and psychosomatic counselling<br>V0 and V12:<br>Classified variables: sex, type 1 diabetes, type 2 diabetes, hypertension, atherosclerotic disease, childhood obesity, sleep apnea syndrome, smoking habits, contraception, medication<br>Numerical variables:<br>age, weight (kg), BMI (kg/m^2), excessive weight (kg), waist/hip ratio, waist (cm), hip (cm), percentage body fat (%) by bioimpedance analysis, fat loss, excessive weight loss, muscle mass (%), caloric uptake (kcal/d), diabetes duration, systolic and diastolic blood pressure (mmHG), glucose (mg/dl), C-peptide (ng/ml), insulin (mU/l), HbA1c (%), HOMA index (HOMA1-IR), LDL cholesterol (mg/dl), HDL cholesterol

	(mg/dl), total cholesterol (mg/dl), triglycerides (mg/dl), Lp(a) (mg/dl), creatinine (mg/dl), eGFR (ml/min.), CRP (mg/dl)
**Screening procedure and data acquisition**	Patients were regularly seen at the outpatient obesity center at clinical visits (V3: after 3 months; V12: after 12 months). Serum samples were obtained in the fasted state regularly together with an assessment of anthropometric, clinical and psychological data together with medication, smoking habits and nutritional assessment. At every visit, clinical and routine laboratory investigation was done. EDTA samples for genomic DNA extraction were obtained at V0. Serum samples (21.5 ml) at V0 and V12 were obtained in the fasted state. Subcutaneous and visceral adipose tissue samples were obtained during bariatric surgery under sterile conditions.
**Data base and statistical analysis**	The *ROBS* database was programmed with *FileMaker Pro 13*, a relational database management program which runs on Windows and Mac Systems as a multi-user system. An additional web interface can be programmed for database applications on iOs- and android-compatible devices. Pseudonymization runs over a 256 bit encoded database that is separated from the general network. In this system, target appointments for the visits are fixed automatically. A separate database with an individual input mask was programmed for data entry patient visits.
**Table 1**	

In the underlying, perpetual study *ROBS*, n= 235 patients (53 males, 182 females) undergoing bariatric surgery have been included so far (VSG, vertical sleeve gastrectomy: n=48; RYGB, Roux-en-Y gastric bypass: n=187). In a parallel group, n=179 patients (52 males, 127 females) undergoing low calory diet (LCD) have been included so far.
